# Icariin, a Multi-Target Natural Neuroprotectant, and a Comprehensive Review on Its Synthetic Methods, Pharmacological Evaluation and Pharmacokinetic Characteristics

**DOI:** 10.3390/ijms27219562

**Published:** 2026-10-26

**Authors:** Kecan Zhang, Jiaxin Li, Weikang Ban, Pinqiu Chen, Xue Ding, Lingjuan Lv, Baolin Guo, Zhihong Yang

**Affiliations:** 1 Institute of Medicinal Plant Development, Chinese Academy of Medical Sciences & Peking Union Medical College, Beijing 100193, China; zhangkecan11@163.com (K.Z.); s2025009019@student.pumc.edu.cn (J.L.); bwk990813@163.com (W.B.); 15695197298@163.com (P.C.); ding0911xue@163.com (X.D.); ljlv1998@163.com (L.L.);; 2 National Engineering Laboratory for Breeding of Endangered Medicinal Materials, Beijing 100193, China

**Keywords:** icariin, synthesis, brain protection effect, pharmacokinetics characteristics, research progress

## Abstract

Epimedii Folium (Yinyanghuo), a unique and traditional Chinese medicinal plant used in medicine for more than a thousand years, has been traditionally used to tonify the liver and kidneys, strengthen the muscles and bones, and eliminate rheumatism. Its principal active ingredient, icariin, has long been used clinically for impotence and spermatorrhea, weakness of muscles and bones, rheumatism and arthralgia pain, and has recently attracted growing attention for its therapeutic potential against central nervous system diseases. This review summarizes advances over the past five years in the preparation, neuroprotective effects, and pharmacokinetics of icariin. The literature was retrieved from Pubmed, CNKI (China National Knowledge Infrastructure), Web of Science, and Google Scholar using keywords including “icariin”, “Epimedii Folium”, “neuroprotection”, “biosynthesis” and “pharmacokinetics”, and studies were screened for relevance to preparation methods, pharmacological evaluation, and pharmacokinetic properties. Although extraction from plant sources remains the main preparation method, chemical synthesis and biosynthesis are emerging in response to resource scarcity. Accumulating evidence indicates that icariin exerts neuroprotective effects through multiple mechanisms, including anti-neuroinflammation, mitochondrial protection, antioxidant effects, inhibition of neuronal apoptosis, promotion of neuronal proliferation and differentiation and regulation of synaptic plasticity. Its rapid absorption, ability to cross the blood–brain barrier, and low cumulative toxicity provide a favorable pharmacokinetic basis for central nervous system applications. This review highlights synthetic strategies, pharmacological evaluation and pharmacokinetic characteristics of icariin, aiming to support its broader application in CNSDs.

## 1. Introduction

Epimedii Folium (EF), the dried leaves of the *Epimedium* species, is a traditional Chinese medicinal herb also known as Yinyanghuo, derived from perennial herbs of the family Berberidaceae [1]. EF was first recorded in the Shennong’s Classic of Materia Medica and has been used for over two thousand years to invigorate kidney-Yang, strengthen muscles and bones, and dispel rheumatism [2]. Modern studies have shown that EF contributes to the preventive and therapeutic benefits in various diseases such as infertility, myocardial infarction, osteoporosis, and—more recently—central nervous system diseases such as ischemic stroke and depression [3,4,5,6,7,8]. As a medicinal and food homology substance, EF is also widely used in health products. There are over 500 healthy foods containing EF currently available on the market, including Epimedium and Ginseng Chewable Tablets, Epimedium and Ginseng Taurine Capsules, etc., which can be found at the State Administration for Market Regulation [9]. It is usually combined with *Cinnamomi* Cortex, *Lycii* Fructus and other herbs that tonify the spleen and augment qi, serving as a formula for health food products [10].

EF contains various pharmacological components [11], including flavonoid compounds such as icariin, epimedin A, epimedin B, epimedin C, and icaritin, the hydrolysis product of icariin; polysaccharides; alkaloids such as magnoflorine; and lignan and terpene compounds. These components confer a wide range of pharmacological effects, such as promoting osteoblast differentiation and improving osteoporosis [12,13]; enhancing the functions of the reproductive system [14] and cardiovascular system [15]; protecting the kidney [16,17]; and exerting anti-inflammatory and anti-tumor activities [11]. Notably, EF also demonstrates neuroprotective effects in the treatment of central nervous system diseases [18].

**Figure 1:**
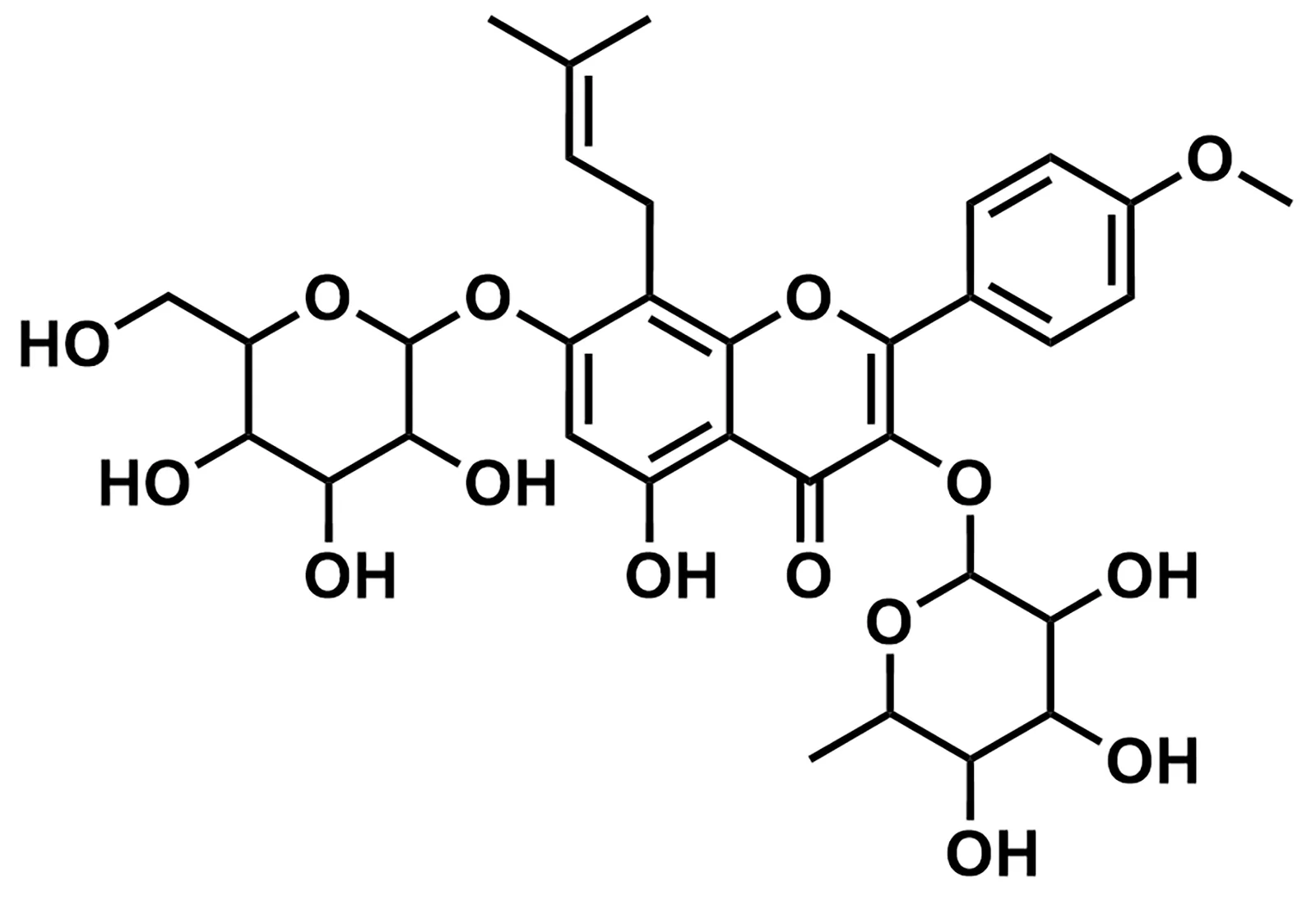
Chemical structure of ICA (C_33_H_40_O_15_).

Icariin (ICA), a 3,7-diglycosylflavone and the most representative 8-prenylflavonoid glycoside in EF (Figure 1), is the major pharmacologically active ingredient of EF and an indicator component for its quality control in the Pharmacopeia of the People’s Republic of China [19]. Owing to its diverse pharmacological activities, ICA has received extensive attention. ICA exhibits anti-inflammatory, antioxidant, anti-apoptotic and lipid metabolism-regulating activities, providing a pharmacological basis for its therapeutic potential against Alzheimer’s disease (AD) [20], rheumatoid arthritis [21], cardiovascular diseases [22,23], ulcerative colitis [24] and other diseases.

Central nervous system disorders (CNSDs) [25,26] are a heterogeneous group of multifactorial conditions characterized by progressive neuronal degeneration and synaptic dysfunction. Major subtypes include AD, Parkinson’s disease (PD), major depressive disorder, and epilepsy, which share common pathological features such as neuroinflammation, oxidative stress, and impaired protein homeostasis. Given their substantial burden of disability, and socioeconomic impact, CNSDs represent a critical public health priority worldwide. Notably, ICA exerts significant protective effects on the central nervous system (CNS) through mechanisms including mitochondrial protection, regulation of neurotrophic factors and neurotransmitters, and reduction in neurotoxic proteins, thereby preventing and treating neurodegenerative diseases [27,28].

With its potential to act on multiple targets and pathways, ICA is emerging as a promising candidate for the treatment of CNS diseases. This review systematically summarizes the preparation methods of ICA, its in vitro and in vivo biological activities against CNS diseases, and its pharmacokinetic characteristics, aiming to provide a theoretical basis for the broader application of ICA in CNSDs.

## 2. Method

The relevant literature on icariin published up to July 2026 was retrieved from multiple databases, including PubMed(National Center for Biotechnology Information, Bethesda, MD, USA), CNKI (China National Knowledge Infrastructure, Beijing, China), Web of Science (Clarivate, Philadelphia, PA, USA), and Google Scholar (Google LLC, Mountain View, CA, USA). Studies addressing the biosynthesis of icariin, its neuroprotection effect and its pharmacokinetics were included and analyzed.

## 3. Preparation Methods of ICA

The preparation methods for ICA include extraction, chemical synthesis, and biosynthesis, with extraction remaining the primary approach. However, due to the low natural yield of ICA, the heavy use of organic solvents and the inability of extraction to meet market demand, researchers are exploring chemical and biological alternatives to improve efficiency, environmental friendliness and sustainability.

### 3.1. Extraction Methods of ICA

The structure of the compound determines its physical properties, which in turn determine the extraction method. As a flavonoid glycoside, ICA contains one glucose molecule and one rhamnose molecule and is soluble in ethanol and ethyl acetate but insoluble in ether, benzene, and chloroform. It is often extracted with ethanol of appropriate concentration. The 2025 edition of the *Chinese Pharmacopeia* (Volume I) clearly stipulates that ICA is extracted from fresh EF with 20 times the amount of ethanol, and the total flavonoids in the leaves are calculated as ICA and shall not be less than 5.0%. Studies show that the extraction rate of ICA is affected by the time of boiling and extraction, the amount of solvent, and the degree of crushing of EF leaves, and the yield of ICA varied from 0.32% to 0.58% [29].

The researchers explored and optimized the extraction methods of ICA from multiple perspectives. During the extraction process of EF, there are complex chemical transformations. High temperature promotes the transformation of ICA, with a transfer rate reaching 301.38%, which is 2.38 times that under the extraction condition of 70% ethanol [30]. The traditional hot water extraction method was also applied to the extraction of EF total flavonoids [31]. Compared with ethanol as the solvent, hot water extraction is simple, low-cost and environmentally friendly, but ICA cannot be selectively extracted. The new technology “ultrasonic-assisted extraction” (UAE) method has been applied to the extraction of ICA, which can extract ICA efficiently, and the yield of ICA extraction has reached 12.85% [32]. Currently, the primary means of acquisition of ICA is still chemical isolation from fresh or dried EF leaves, which is simple to operate. However, it has the drawbacks of low yield and high organic solvent consumption, making it less environmentally friendly.

### 3.2. Synthesis Method of ICA

Owing to harsh growing conditions, habitat loss, and the low yield of ICA from EF, the natural resources of ICA have declined markedly in recent years [33]. Consequently, chemical synthesis and biosynthesis of ICA have emerged as promising alternatives, laying the foundation for the conservation and sustainable utilization of EF.

#### 3.2.1. Chemical Synthesis

**Scheme 1:**
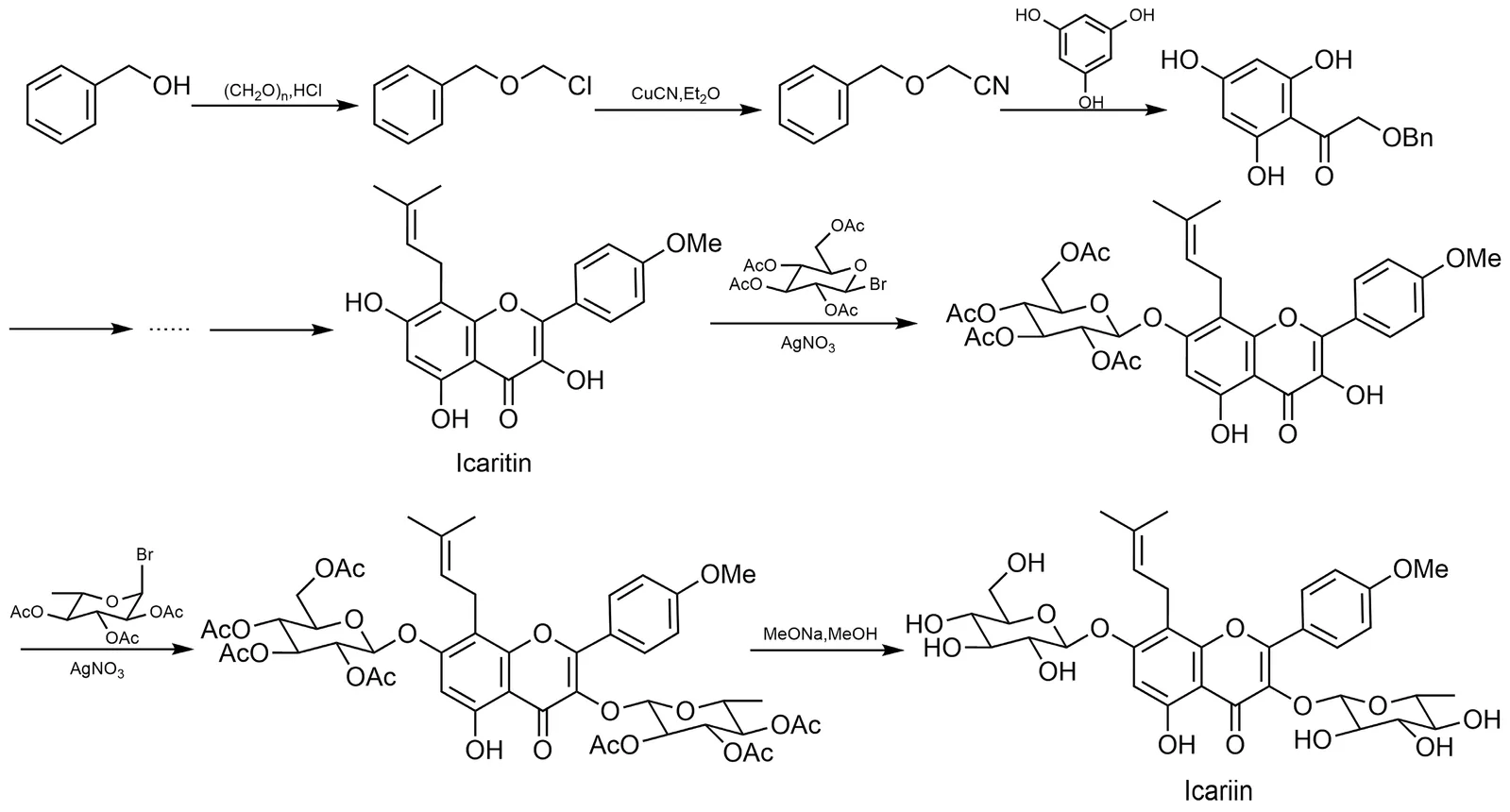
The total synthesis method of ICA from benzyl alcohol and phloroglucinol.

ICA was totally synthesized from benzyl alcohol and phloroglucinol with an overall yield of 0.6%, in which icaritin (ICT) served as the intermediate (Scheme 1) [34]. This technology uses readily available raw materials. However, it involves as many as 15 tedious steps and a low overall yield, which hinders its industrial application. In another study, ICA was synthesized directly from ICT as the starting material, increasing the yield to 47.78% [35]. The reaction followed the same route as Scheme 1, differing only in the catalyst. Although this method improved the yield, it was also not applicable to industrial production due to the high price of raw material ICT.

**Scheme 2:**
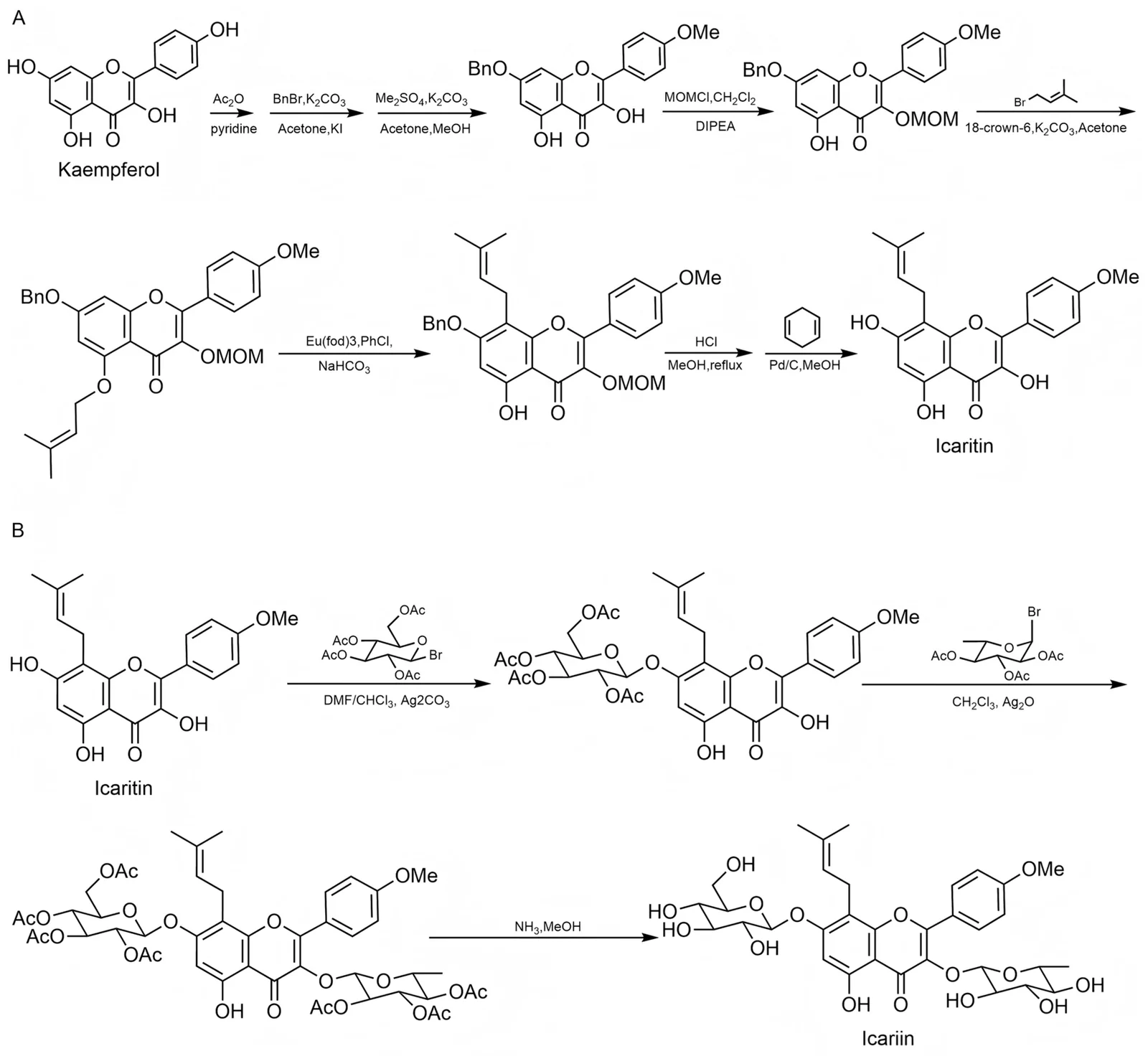
Method for chemical synthesis of ICA by kaempferol. (**A**) The chemical synthesis of intermediate ICT. (**B**) The chemical synthesis of ICA.

The semisynthesis of ICA is mainly based on commercially available kaempferol. In one study, starting from the preparation of its aglycone ICT, ICA was obtained in 11 semisynthetic steps. Specifically, kaempferol underwent acetylation, benzylation, deethylation, methyletheration, O-pentenylation, Claisen rearrangement and deprotection, affording ICT in 26% yield (Scheme 2A). Subsequently, 7-OH and 3-OH glycosylation of ICT were performed with tetra-O-acetylglucopyranosyl bromide and tri-O-acetylrhamnopyranosyl bromide respectively, and then all acetyl groups were removed to obtain ICA, with 7% overall yields (Scheme 2B) [36]. Derivatives of ICA and other structurally related flavonoid analogs, such as icariside I (ICS I), can also be prepared using this method.

Although chemical synthesis is a feasible means of obtaining ICA, it still suffers from drawbacks such as harsh reaction conditions, numerous by-products and low yields.

#### 3.2.2. Biological Synthesis

**Scheme 3:**
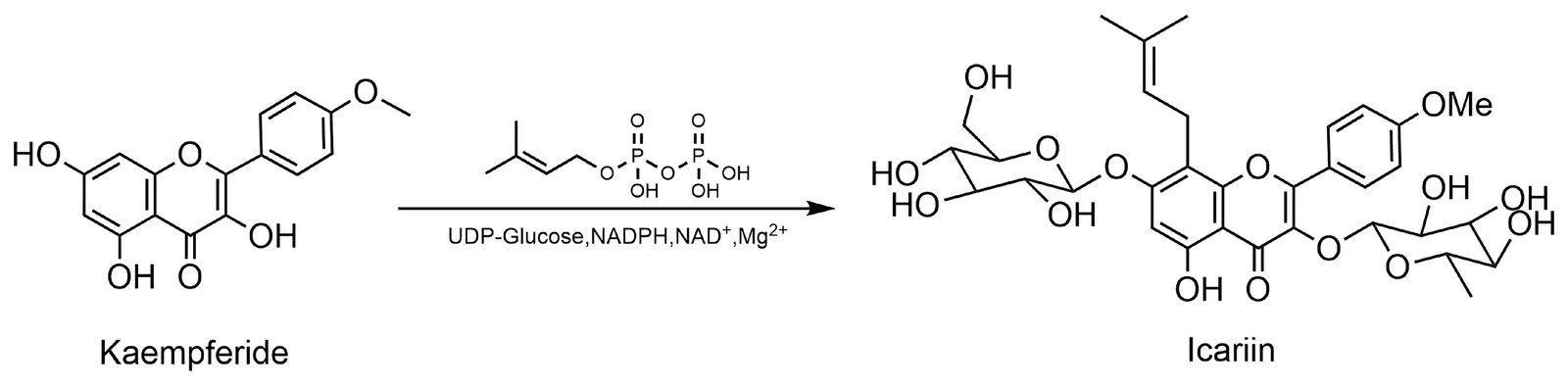
Method for biological synthesis of ICA by kaempferide in one step.

**Scheme 4:**
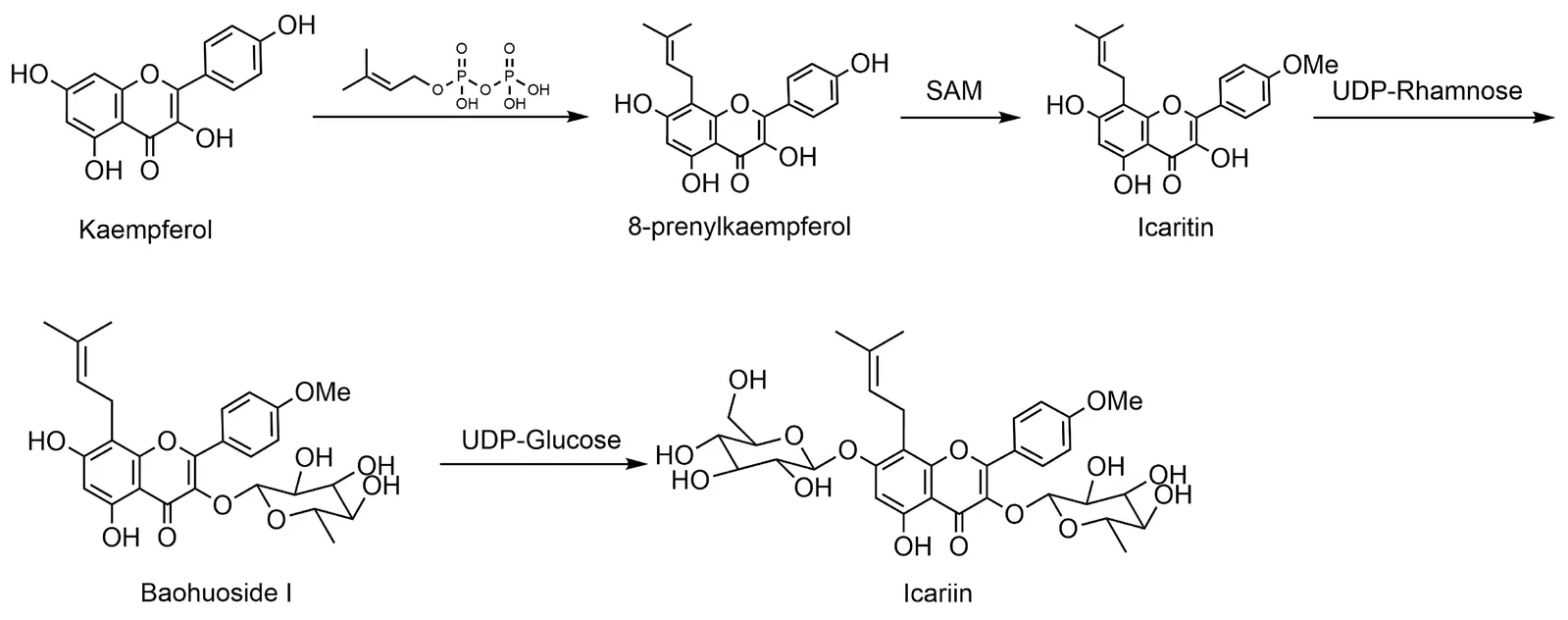
Method for biological synthesis of ICA by kaempferol.

Biosynthetic ICA possesses more advantages, including fewer by-products, milder reaction conditions and less pollution, compared with those derived from the traditional extraction method and chemical synthesis methods. It has been reported that ICA was biosynthesized in a one-step enzyme-catalyzed reaction using kaempferide as the substrate (Scheme 3). Feng et al. [37] established a system containing enzyme SfFPT (8-prenyltransferase), EpRhS (transfer UDP—glucose to UDP—rhamnose), EpPF3RT, and Ep7GT (glycosyltransferases). Under this system, the mixture of kaempferide, DMAPP and UDP—glucose was reacted at 30 °C for 12 h, resulting in ICA and a conversion rate of 5%. Researchers have successfully constructed and optimized the biosynthetic pathway of ICA in *Saccharomyces cerevisiae*, achieving de novo synthesis of ICA from glucose. The heterogeneous flavonoid biosynthesis pathway was introduced to engineer *Saccharomyces cerevisiae* to produce kaempferol—the precursor for the biosynthesis of ICA—which subsequently synthesized ICA with a final titer of 130 μg/L by 8-C-prenylation, 4′-O-methylation, 3-O-rhamnosylation and 7-O-glucosylation (Scheme 4) [38].

**Scheme 5:**
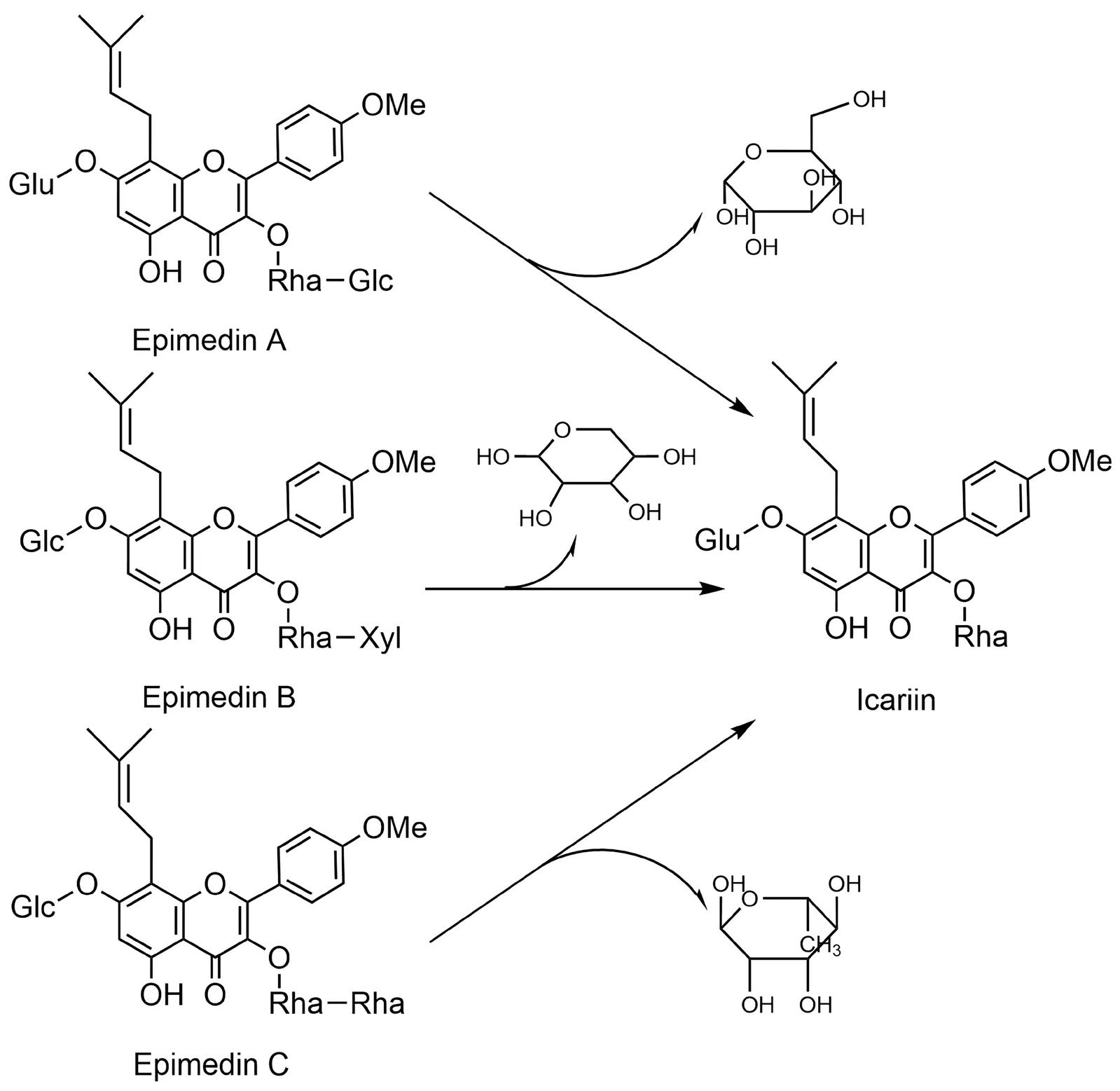
Method for biological synthesis of ICA by epimedins.

Flavonoids are the main active components of EF, including epimedin A, epimedin B, epimedin C and ICA. These compounds have similar structures and are characterized by isopentenyl substitution at C_8_ [39]. The similarity of structures provides the possibility of transforming epimedins into ICA (Scheme 5) [33]. Studies have shown that an α-L-rhamnosidase isolated from *Aspergillus nidulans* [40] and *Papiliotrema laurentii* ZJU-L07 [41] could convert epimedin C into ICA under specific conditions, with conversion efficiencies of 100% and 83%, respectively. Novel GH78 α-L-rhamnosidase AmRha [42] and BtRha [43] could transform epimedin C to ICA by 92.3% and 90.5%, respectively. Compared with the ethanol extraction method, the biosynthesis of ICA can increase the yield by more than 50%. However, the reaction time needs to be paid special attention to; too long a reaction time will further hydrolyze the sugar group to get ICS or ICT [44].

The biological synthesis of ICA not only causes less pollution and requires milder reaction conditions, but can also make up for the shortcomings of the low yield of the traditional extraction method and complicated steps of the chemical synthesis method. The obtained enzymes are active and stable in conditions that could meet industrial applications and be used for the industrialized production of ICA.

## 4. Central Nervous System Protection

A large number of pharmacological studies have proved that ICA has effects in preventing and treating depression, PD, AD, cerebral ischemia and multiple sclerosis. It can also delay aging and improve learning and memory, thereby protecting the CNS [45,46].

**Figure 2:**
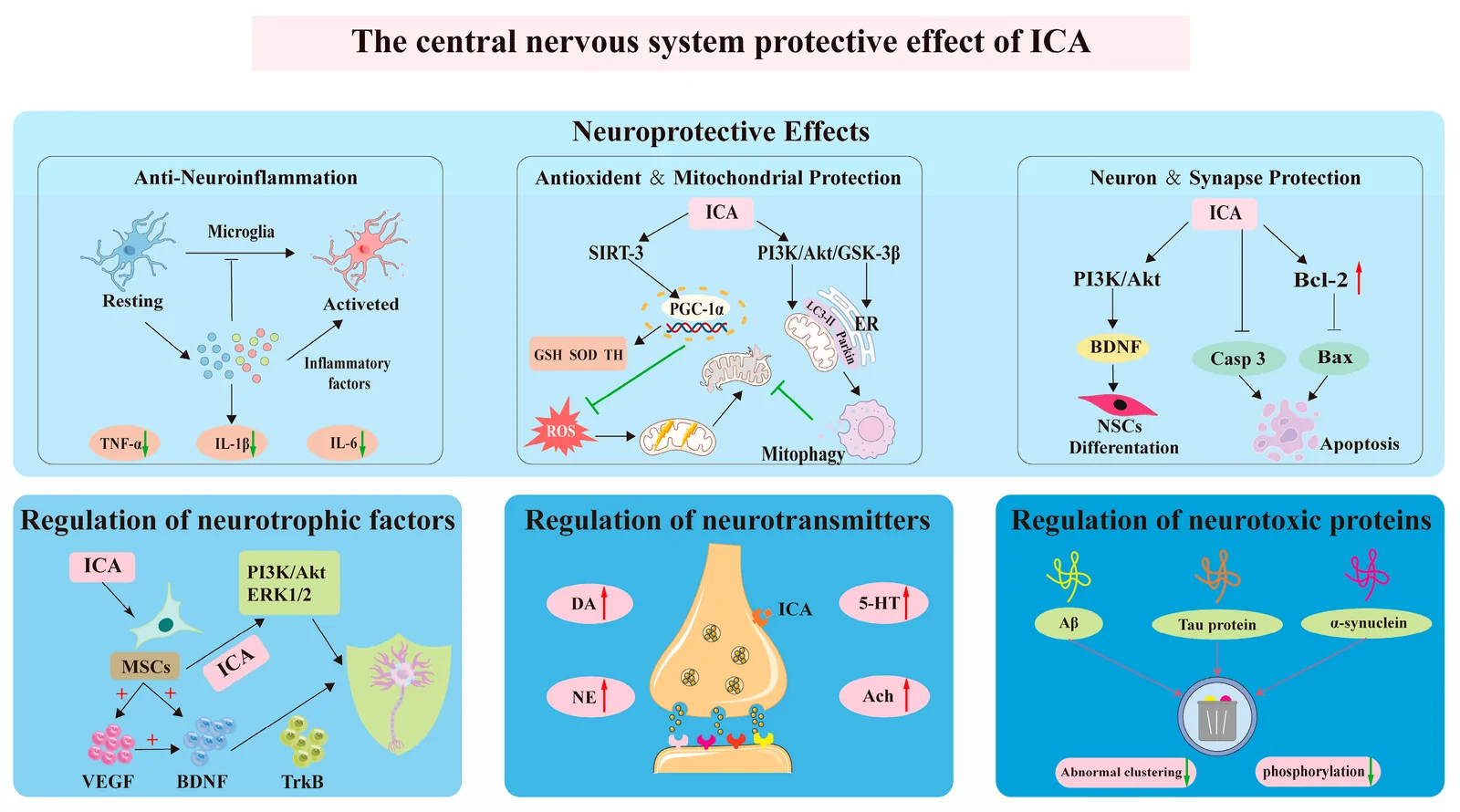
Six possible mechanisms of ICA’s protective effect on central nervous system. Each part (mechanism) of the figure will be discussed in the manuscript in the following pages. Red arrows represent increased expression of the related proteins, whereas green arrows represent decreased expression. The red plus sign indicates enhanced production of the related factors. TNF-α, tumor necrosis factor-α; IL-6, interleukin-6; IL-1β, interleukin-1β; ICA, icariin; GSH, glutathione; GSK-3β, glycogen synthase kinase-3 beta; ROS, reactive oxygen species; MDA, malondialdehyde; SOD, superoxide dismutase; BDNF, brain-derived neurotrophic factor; VEGF, vascular endothelial growth factor; DA, dopamine; NE, norepinephrine; 5-HT, 5-hydroxytryptamine; ACh, acetylcholine; Aβ, amyloid β-protein; Casp 3, caspase-3; Bax, Bcl-2-associated X protein; TrkB, tropomyosin receptor kinase B; Bcl-2, B-cell lymphoma 2; PI3K/Akt, phosphoinositide 3-kinase/protein kinase B; MSCs, mesenchymal stem cells; ERK1/2, extracellular signal-regulated kinase 1/2.

In vivo and in vitro experiments have shown that ICA can reduce cell function damage by improving oxidative stress damage and inhibiting inflammation. ICA can exert neuroprotective effects, which are achieved through protecting mitochondria, remodeling the formation and plasticity of synapses, promoting the proliferation of nerve cells and inhibiting cell apoptosis. In addition, ICA can also regulate the release of neurotrophic factors, and it can protect the CNS by releasing neurotransmitters and anti-neurotoxic substances to prevent damage (Figure 2). In recent years, the protective effect of ICA on the CNS has received widespread attention. Here, the pharmacological development of ICA and its possible mechanism in the prevention and treatment of CNS diseases are reviewed and summarized, with a view toward expanding the understanding of the preventive and therapeutic role of ICA in neurodegenerative diseases.

### 4.1. Neuroprotective Effects

ICA exerts neuroprotective effects through a variety of pathways, including anti-neuroinflammatory and antioxidant effects, protection of mitochondria and neurons, and regulation of synaptic formation and plasticity (Table 1).

#### 4.1.1. Anti-Neuroinflammation

The inflammatory response involves interactions between the CNS and peripheral immunity. The activation of intravascular inflammatory response can cause neuronal injury through the release of neurotoxic cytokines and inflammatory mediators. ICA can improve immune organ function, inhibit a variety of inflammatory factors to reduce inflammation in the CNS. Cao et al. [4] found continuous intragastric administration of ICA (12.5, 25, 50 mg/kg) for 30 days significantly increased the organ indexes of spleen and thymus in rats with the perimenopausal depression-like model. In various depression models, ICA could promote learning and memory abilities by regulating the expression of tumor necrosis factor-α (TNF-α), interleukin-6 (IL-6), IL-1β, IL-10 in the hippocampal CA1 region or serum [56,57], thereby further alleviating the neuroinflammation response. In the experiment of pilocarpine model of epilepsy mice, it was observed that the intensity and mortality of acute epileptic mice and the release of pro-inflammatory factor IL-1β, IL-6 was reduced after ICA intervention. Flow cytometry showed that pre-administration of ICA substantially decreased the microglial polarization M1/M2 phenotype ratio, indicating that ICA plays an anti-epileptic role by promoting M2 polarization of microglia [58].

Multiple signaling pathways play important roles in the improvement of neuroinflammation by ICA. ICA can effectively inhibit neuroinflammation by attenuating microglia and astroglia activation, through enhancing nuclear factor erythroid 2-related factor 2 (Nrf2) signing activation [59,60,61]. Further experiments showed that ICA could also reduce the expression of NLR family pyrin domain-containing 3 (NLRP3) in downstream targets of nuclear factor kappa B (NF-κB) signaling pathway, and inhibit the expression of apoptosis-associated speck-like protein containing a CARD (ASC) and cysteine-dependent aspartate-specific protease 1 (Caspase-1), thus treating cerebral ischemia–reperfusion injury [62]. In vivo, ICA can inhibit the HMGB1-RAGE signaling pathway and activate the TLR4-XBP1s-related NF-κB signaling at the same time, thereby improving hippocampal neuroinflammation and demonstrating neuroprotective effects [63]. In vitro, Mo et al. discovered that by administering ICA, the IRE1/XBP1s pathway could be inhibited, thereby achieving the regulation of the protein expression levels of IL-1β, IL-6 and TNF-α in microglia cells subjected to oxygen–glucose deprivation and reoxygenation (OGD/R) injury [64]. Collectively, these findings indicate that ICA ameliorates neuroinflammation by protecting immune organs, regulating inflammatory factors and inhibiting the activation of microglia through various signaling pathways.

Renal encephalopathy (RE) is a disease characterized mainly by cognitive dysfunction due to renal insufficiency. At present, effective therapeutic methods remain limited. Research has shown that ICA can effectively improve cognitive dysfunction and renal function in RE mouse models by regulating NF-κB, Nrf2 and apoptosis pathways [65].

#### 4.1.2. Anti-Oxidative Stress and Mitochondria Protection

Damage to the CNS can lead to an excessive production of reactive oxygen species (ROS). Oxidative stress occurs when the scavenging capacity of the antioxidant system is exceeded. The role of ICA in inhibiting oxidative stress has been demonstrated in brain injury models induced by different chemical agents. Zhou et al. treated D-galactose-induced brain aging model mice with ICA (75, 150 mg/kg) for 10 weeks. This treatment significantly increased total antioxidant capacity (T-AOC), glutathione peroxidase (GSH-Px) and glutathione (GSH) levels, while significantly reducing the content of malondialdehyde (MDA) [66]. Identically, in haloperidol-induced PD in rats, ICA exerted neuroprotective effects by decreasing neurotoxicity through lowering midbrain lipid peroxidation MDA, NO content and increasing antioxidant biomarkers tyrosine hydroxylase (TH), glutathione S-transferases (GST), catalase (CAT) and superoxide dismutase (SOD) [67]. So, ICA can treat CNS diseases by regulating levels of antioxidant biomarkers, suggesting that it is a promising candidate medicine for antioxidant therapy in CNS diseases.

As organelles related to energy metabolism, mitochondria play an important role in the production of ROS, autophagy and calcium signaling. Therefore, drugs targeting CNS diseases must exert mitochondrial protection. ICA could activate the genes and proteins related to autophagy in cortical mitochondria to exert mitochondrial protection. Treatment with ICA (75, 150 mg/kg, 4 weeks) ameliorated mental state, diet and activity in D-galactose-induced brain aging mice and increased the expression of key mitochondrial autophagy proteins, including LC3-II, Parkin, and Bnip3L in the cortex [45]. The amyloid β-protein (Aβ) plays a crucial role in the onset of AD, and ICA has been found to inhibit Aβ-induced mitochondrial damage by enhancing PKB/Akt phosphorylation [68]. Administration of ICA either before or after Aβ_1–42_ injection in rats significantly attenuated autophagic–lysosomal dysfunction, as evidenced by a reduced LC3-II/LC3-I ratio, decreased Beclin-1 protein expression, and lower Cathepsin D levels [68]. Also, ICA can increase the mitochondrial membrane potential of schizophrenia model rats injected with the *N*-methyl-D-aspartate receptor antagonist, MK-801, by regulating the NRG1/ErbB4 signaling pathway [69]. In chronic unpredictable mild stress (CUMS) mouse models, ICA can protect mitochondria by inhibiting mitochondrial GR translocation and reducing mitochondrial outer membrane permeabilization (MOMP) [70]. All in all, these in vivo findings indicate that ICA regulates relevant signaling pathways, modulates mitochondrial autophagy, and reduces mitochondrial damage, thereby exerting mitochondrial protection.

In vitro experiments have confirmed that ICA can reduce Ca^2+^ concentration and thus play a key role in protecting mitochondria. ICA (1–100 μM) inhibited the increase in mitochondrial membrane potential by reducing intracellular ROS, NO levels and Ca^2+^ concentrations, and protected SH-SY5Y against glutamate-induced excitotoxicity [71]. Similar results were obtained by isolating neural cells from neonatal rats and simulating ischemia–reperfusion (I/R) injury with OGD/R, found that ICA reduced the increase in ROS, lactate dehydrogenase (LDH) and Ca^2+^ concentration in OGD/R cells, thereby protecting the neural cells from I/R injury [72]. Studies have proved that ICA can protect PC12 cells through several mechanisms. One study has certified that ICA promotes mitochondrial respiration and reduces ROS generation by elevating the protein expression of peroxisome proliferator-activated receptor gamma coactivator 1-alpha (PGC-1α) through the mediation of sirtuin 3 (SIRT3) in PC12 cells [73]. Another study confirmed that ICA (0.01–1 μM) reduced LDH leakage, increased mitochondrial membrane potential, and restored energy metabolism in NaN3-induced neuronal PC12 cells by activating the PI3K/Akt/GSK-3β signaling pathway [74]. Multiple in vitro experiments have demonstrated that ICA can regulate mitochondrial membrane potential, maintain mitochondrial respiration, and ensure the energy metabolism of cells.

Overall, in vivo and in vitro experiments have shown that ICA protects mitochondria, regulates a variety of oxidative stress markers such as SOD, MDA, and GSH, and can reduce neurotoxicity and protect nerve cells through antioxidant effects.

#### 4.1.3. Neurons and Synapses Protection

The injury of the CNS leads to the activation of inflammation and the secretion of a large number of inflammatory factors, which induce the apoptosis of nerve cells [75]. Multiple studies have shown that ICA can effectively alleviate this kind of damage. Researchers found that ICA reduced neuronal apoptosis by up-regulating the expression of Bcl-2 and down-regulating the expression of Bax and caspase-3 in the cerebral cortex of middle cerebral artery occlusion (MCAO) rats [57,76,77]. Compared with CUMS rats, treatment with ICA (120 mg/kg, 6 weeks) resulted in a significant increase in the number of neurons (*p* < 0.01) in the dentate gyrus and promoted proliferation and differentiation of hippocampal neural stem cells. Cerebrospinal fluid proteomic analysis revealed that PI3K/Akt and IL-17 signaling pathways might be involved [78]. The PI3K/Akt signaling pathway might also be one of the mechanisms through which ICA reduces cerebral edema and neuronal apoptosis in rats with cerebral ischemia–reperfusion injury [79]. In vitro, ICA (0, 50, 100 µM) could increase the cell cycle gene and protein expression of cyclin D1 and p21, and promote the growth and proliferation of neural stem cells from the rat hippocampus in a dose-dependent manner [80]. ICA inhibited neuronal apoptosis and promoted the proliferation and differentiation of neural stem cells through several signaling pathways.

In neurodegenerative diseases, synaptic loss accompanied by functional decline is one of the main features [81], and ICA regulates synaptic formation and plasticity. ICA (60 mg/kg, 4 weeks) could enhance the density of dendritic spines and the thickness of postsynaptic density, and up-regulate the expression of postsynaptic-associated proteins such as postsynaptic density protein 95 (PSD-95) and *N*-methyl-D-aspartate receptor subunit, glutamate ionotropic receptor NMDA type subunit 1 (GluN1) in the hippocampus of senile dementia model rats [82]. ICA (30, 60, 120 mg/kg, 21 days) significantly reversed the reduced synaptic density, slow development and poor plasticity in the CA1 region of the hippocampus in a dose-dependent manner, and up-regulated synapse-associated proteins, including growth-associated protein 43, PSD-95, and synaptophysin in Aβ_1-42_-induced AD mice through the brain-derived neurotrophic factor (BDNF)-tropomyosin receptor kinase B (TrkB) signaling pathway [83]. Another experiment also proved that ICA ameliorated synaptic plasticity, as evidenced by increased synaptic number, cleft curvature, active zone and proportion of perforated synapses, as well as decreased synaptic cleft width, in a positive dose–effect relationship in the CA1 region of Aβ_1-42_-infused rats through the BDNF/TrkB/Akt pathway [84].

In summary, ICA can inhibit neuronal apoptosis, promote neuronal proliferation and differentiation after CNS injury through various mechanisms, and regulate synaptic formation and plasticity.

### 4.2. Regulation of Neurotrophic Factors

ICA stimulates the secretion of BDNF and vascular endothelial growth factor (VEGF), thereby facilitating the repair of damaged neurons and the formation of new blood vessels, and exerting positive effects on nerve function protection.

#### 4.2.1. Brain-Derived Neurotrophic Factor

BDNF is a neurotrophic factor widely expressed in the CNS, and regulates synaptic and axonal plasticity associated with learning, memory, and sensorimotor recovery [85]. BDNF works together with TrkB to nourish nerve cells and enhance learning and memory abilities, and researchers have observed decreased BDNF levels in a variety of neurodegenerative diseases [86]. Sheng et al. have found that oral administration of Bushen-Tiansui Formula (ICA is one of the main active ingredients) can reverse the cognitive defects in an Aβ_1-42_ fibril-infused rat model of AD, which could protect synaptic ultrastructure in the CA1 region and restore the expression of BDNF and synaptotagmin through activation of the BDNF receptor TrkB and the downstream Akt/CREB signaling pathway [87]. ICA (200, 400, 800 mg/kg, 8 weeks) improved the spatial memory ability of vascular dementia rats, enhanced the expression of BDNF in the hippocampus, and reduced neuronal degeneration, necrosis and apoptosis. The mechanism may be that ICA activates the downstream ERK/CREB signaling pathway through the BDNF-TrkB signaling pathway [88]. In addition, in schizophrenic rats, ICA (30 mg/kg, 2 weeks) increased the expression of BDNF, TrkB, protein kinase C (PKC), Raf-1 and ERK1/2 by activating BDNF/TrkB-mediated PKC/Raf-1 and extracellular signal-regulated kinase (ERK) signaling pathways [89]. ICA (30, 60, 120 mg/kg, 3 weeks) can reduce the content of Aβ_1-42_ in the hippocampus of AD mice, repair neuronal damage, enhance synaptic plasticity, and increase the expression of BDNF, TrkB, cAMP-response element binding protein (CREB), Akt, GAP-43, PSD-95 and SYN proteins in the hippocampus in a dose-dependent manner [83]. ICA up-regulates the expression of BDNF in the brain through multiple signaling pathways, thereby protecting the CNS.

#### 4.2.2. Vascular Endothelial Growth Factor

VEGF is the most potent mitogen for vascular endothelial cells, driving their proliferation and angiogenesis—processes critically implicated in neuroprotection, as well as the modulation of learning and memory functions [90]. ICA (15, 30, or 60 mg/kg, 4 weeks) could promote angiogenesis after chronic cortical hypoperfusion in rats by up-regulating VEGF and decreasing NO concentration in the cerebral cortex [91]. In experimental rats with focal cerebral ischemia treated with ICA combined with mesenchymal stem cell (MSC) transplantation, it was observed that administering a dose of 60 mg/kg of ICA for 28 consecutive days was significantly more effective than the MSC transplantation therapy alone. It could more effectively reduce the infarct volume in rats with cerebral ischemia and promote the differentiation of neurons. Also, in vivo, ICA obviously increased VEGF production in the ischemic frontal cortex; in vitro, it further promoted BDNF secretion of MSCs. Results demonstrated that ICA and MSCs exerted a synergistic effect on cerebral ischemia treatment through the PI3K/Akt and ERK1/2 pathways [92].

BDNF and VEGF are common neurotrophic factors, and their decreased levels indicate CNS injury. ICA up-regulates the levels of BDNF and VEGF, thereby playing a neuroprotective role.

### 4.3. Regulation of Neurotransmitters

The damage to the nervous system is mainly manifested as a decline in learning and memory abilities. Neurological diseases can lead to changes in brain neurotransmitters, thereby causing neuronal apoptosis and cell degeneration [93]. Studies have reported that ICA can improve brain pathology changes and depressive symptoms in various rodent models by regulating monoamine and cholinergic neurotransmitters.

#### 4.3.1. Monoamine Neurotransmitters

Norepinephrine (NE), dopamine (DA), and 5-hydroxytryptamine (5-HT), as monoamine neurotransmitters, are closely related to the CNS. Numerous studies have demonstrated that ICA enhances rat behavior by increasing the levels of monoamine neurotransmitters. In a PD rat model induced by the dopaminergic D2 antagonist haloperidol, ICA (100 mg/kg, 2 weeks) alleviated neurotoxicity by increasing the levels of NE, DA and 5-HT in the cortex and midbrain [67]. After using ICA (10, 20, and 40 mg/kg, ig., 21 days), the levels of NE, DA, and 5-HT in the prefrontal cortex, hippocampus, and striatum of CUMS rats were significantly increased (*p* < 0.05) [94]. This effect may be achieved through the BDNF-TrkB signaling pathway [95]. ICA (50, 100, 200 mg/kg, 1 week) can also be used in insomnia mouse models established by injecting pchlorophenylalanine (PCPA), it can effectively increase the content of GABA and 5-HT, and bring out sedative-hypnotic effects [96]. In vitro experiments showed that ICA (0.1–10 μM) enhanced the release of NE, DA and 5-HT in PC12 cells following OGD/R. The mechanism underlying this effect may involve reducing Ca^2+^ overload and mitigating cell damage [97]. Up-regulation of monoamine neurotransmitters, such as NE, DA, and 5-HT, may underlie ICA’s effects on improving reduced learning ability and alleviating depression-like behavior associated with CNS injury.

#### 4.3.2. Cholinergic Neurotransmitters

Acetylcholine (ACh) is another type of neurotransmitter closely associated with cognitive, learning and memory functions. It has been reported that ICA and its derivatives can bind to cholinesterase (ChE), thereby inhibiting ACh hydrolysis and maintaining cholinergic function. Moreover, the ICA-ChE complex formed by them can prevent the formation of acetylcholinesterase (AChE), thereby inhibiting the hydrolysis of ACh [98]. In mice with traumatic brain injury, the use of ICA (75–150 mg/kg, 4 weeks) can enhance the acetylation and cholinergic functions of the hippocampus, increasing the content of ACh, enhancing the mRNA and protein expression of choline acetyltransferase (ChAT), and increasing acetylated histone H3 levels, thereby improving cognitive function [99]. Additionally, ICA (15, 30, 60 mg/kg, 4 weeks) modulated the cholinergic system by elevating the levels of ACh and ChAT in the brain, and activated the BDNF/ERK/CREB signaling pathway to improve learning and memory and spatial recognition ability in rats with schizophrenia induced by intraperitoneal injection of the NMDA receptor antagonist MK-801 [100]. Therefore, ICA can enhance acetylation and cholinergic function by inhibiting the decomposition and promoting the synthesis of ACh, thus playing a neuroprotective role.

### 4.4. Reduction in Neurotoxic Proteins

Neurotoxic proteins, including Aβ, tau protein and α- synuclein, are closely related to the pathogenesis and development of AD and PD. ICA can reduce the production of neurotoxic proteins and accelerate their degradation, thereby improving the pathological status of neurodegenerative diseases.

#### 4.4.1. Amyloid β-Protein

The abnormal deposition of Aβ_1-42_ leads to widespread neuronal death and ultimately results in memory loss [101]. ICA can reduce abnormal Aβ deposition in several ways. Researchers found that long-term administration of ICA (60 mg/kg, 8 months) effectively improved cognitive and learning deficits in APP/PS1 mice (AD transgenic mice) and inhibited inflammation by reducing Aβ_40_ and Aβ_42_ deposition [102]. Administration of ICA (60 mg/kg) for 6 months could also reduce M1 phenotype microglia in the hippocampus, the inflammatory response and Aβ accumulation in the hippocampus of APP/PS1 mice [103]. In colchicine-induced cognitive deficit rats, ICA reduced the accumulation of Aβ_1-42_ by up-regulating the levels of neprilysin and insulin-degrading enzyme, and down-regulating the levels of matrix metalloproteinase-2 (MMP2) [104]. ICA inhibited the accumulation of Aβ by reducing the expression of amyloid precursor protein, increasing the expression of ADAM10, a neuroprotective and neurotrophic protein, and stimulating NO-cGMP signaling [105,106,107]. Furthermore, in vitro experiments showed that ICA treatment increased viability and decreased apoptosis of Aβ_25-35_-induced PC12 cells in a dose-dependent manner [108]. ICA also markedly reduced Aβ-induced insulin resistance in human neuroblastoma SK-N-MC cells, which may be helpful in the development of alternative treatments for neurodegenerative diseases [109].

#### 4.4.2. Tau Protein

Hyperphosphorylation of tau protein, which causes neurofibrillary tangle formation, is one of the main pathological changes that occur in AD patients [110]. If a drug can reduce the phosphorylation of tau protein, it suggests that this drug may have the potential to treat AD. Administration of ICA (120 mg/kg) for 4 months led to a decrease in the phosphorylation level of tau protein and Aβ_1-42_ accumulation in the cortex and hippocampus of 3xTg-AD mice [111]. ICA (60 mg/kg, 5 months) was able to modulate brain insulin signaling and glucose transporters to effectively reverse tau hyperphosphorylation [112]. In vitro experiments showed that ICA administration could reverse the increase in p-Tau/Tau and GSK-3β levels in AD model cells [113], and inhibit tau protein hyperphosphorylation at Ser396, Ser404 and Thr205 in Aβ_25-35_-induced PC12 cells by activating the PI3K/Akt signaling pathway [114]. ICA can effectively reduce the excessive phosphorylation of tau protein in several ways, thereby playing a role in treating AD.

#### 4.4.3. α-Synuclein

The formation of Lewy bodies is a typical pathological hallmark of PD; their primary component is α-synuclein, whose accumulation is toxic to cells [115]. In rotenone-induced PD rats, ICA (15, 30 mg/kg, 5 weeks) reduced the accumulation of α-synuclein and alleviated mitochondrial respiration dysfunction [116]. In A53T α-synuclein transgenic (A53T Tg) PD mice, continuous intragastric administration of ICA (33.8, 67.7 mg/kg) for 3 months could significantly improve the impaired motor function and coordination ability of the mice. The mechanism may be related to the regulation of PLK2 and parkin expression, thereby reducing the phosphorylation and aggregation of α-synuclein [117]. In vitro, ICA could significantly reverse the decrease in LC3-II protein expression, the increase in Beclin-1 expression and the activation of mTOR phosphorylation in PC12 cells induced by rotenone, suggesting that ICA exerted neuroprotective effects by enhancing autophagy flux [116]. ICA can reduce the accumulation of α-synuclein in both in vivo and in vitro models, thereby exerting a protective effect on the CNS.

In summary, neurotoxic proteins, as one of the markers of neurodegenerative diseases, play an important role in the disease process. ICA is capable of reducing the deposition of Aβ, tau protein, and α-synuclein, and is associated with multiple signaling pathways, potentially offering a new target for the treatment of neurodegenerative diseases (Table 2).

## 5. Pharmacokinetics

**Figure 3:**
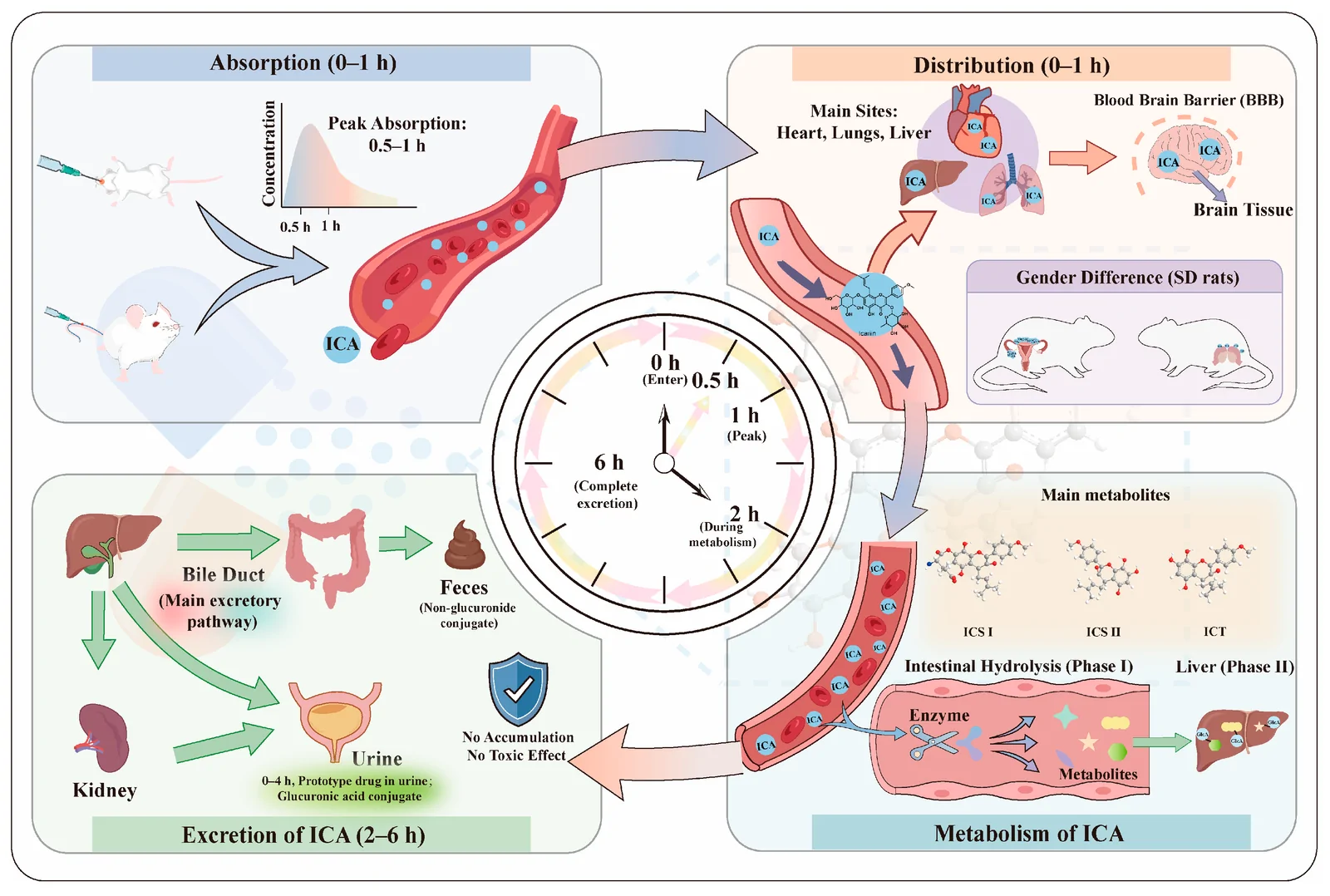
The metabolism process of ICA. This figure illustrates the in vivo pharmacokinetic behavior of ICA, structured according to the four fundamental ADME processes—absorption, distribution, metabolism, and excretion. Each process is characterized by distinct kinetic, distributional, or biotransformation properties, as detailed in the following section.

Pharmacokinetic studies provide a platform for explaining the effectiveness mechanism and fully elucidating the kinetic properties and dynamic changes in drugs in vivo. Since ICA is one of the active components of EF, it is necessary to analyze its metabolic pathways and products. Furthermore, the metabolic process of ICA in the body also has its unique characteristics (Figure 3). The pharmacokinetic parameters of ICA in plasma as a monomer or a drug component are shown in Table 3.

### 5.1. Absorption

The pharmacokinetic characteristics of ICA were reported as early as last century; it conforms to a two-compartment model in both single and compound states, exhibiting rapid absorption rates [141,142]. ICA was detectable in rat plasma as early as 5 min after rats were orally administered 0.69 g/kg of EF total flavonoid extract (containing nearly 42 mg/g of ICA). The time to maximum plasma concentration (T_max_) was 0.458 h, and the AUC was 164.115 μg·h/mL, demonstrating rapid absorption of ICA [143]. Intramuscular injection of Chuankezhi injection, with ICA (approximately 66 μg/kg) as the main component, resulted in a T_max_ of 0.13 h and an AUC of 55.65 ± 9.65 μg·h/mL [144]. Another study showed that after intravenous administration of 30 mg/kg ICA in rats, C_max_ was 3.543 × 10^4^ ng/mL and AUC was 6.942 × 10^5^ ng·h/mL [145]. ICA enters the body quickly, reaching its peak concentration in plasma in approximately 0.5–1 h. However, due to its poor water solubility and membrane permeability, ICA has a bioavailability of only 12.02% [141].

### 5.2. Distribution

ICA can be rapidly and widely distributed throughout the organs and tissues of the whole body in rats about 1 h after administration by gavage or orally. It is mainly distributed in the liver, heart and lungs, and there are gender differences. The content of ICA in the reproductive organs of female rats is significantly higher than that in other tissues except the liver, and also higher than that in the reproductive organs of male rats [143]. Prototype ICA could be detected in brain tissue [146]. The ability of ICA to pass through the blood–brain barrier (BBB) and enter brain tissue is the material basis for its neuroprotective effects [147]. The protein binding rate of ICA in human plasma is about 90% without concentration dependence [148]. In rats, the plasma protein binding rate of ICA was 85% [141]. The high protein binding rate suggests that clinical attention should be paid to drug interactions, and other components in the complex formulation may affect the pharmacokinetic characteristics of ICA.

### 5.3. Metabolism

**Figure 4:**
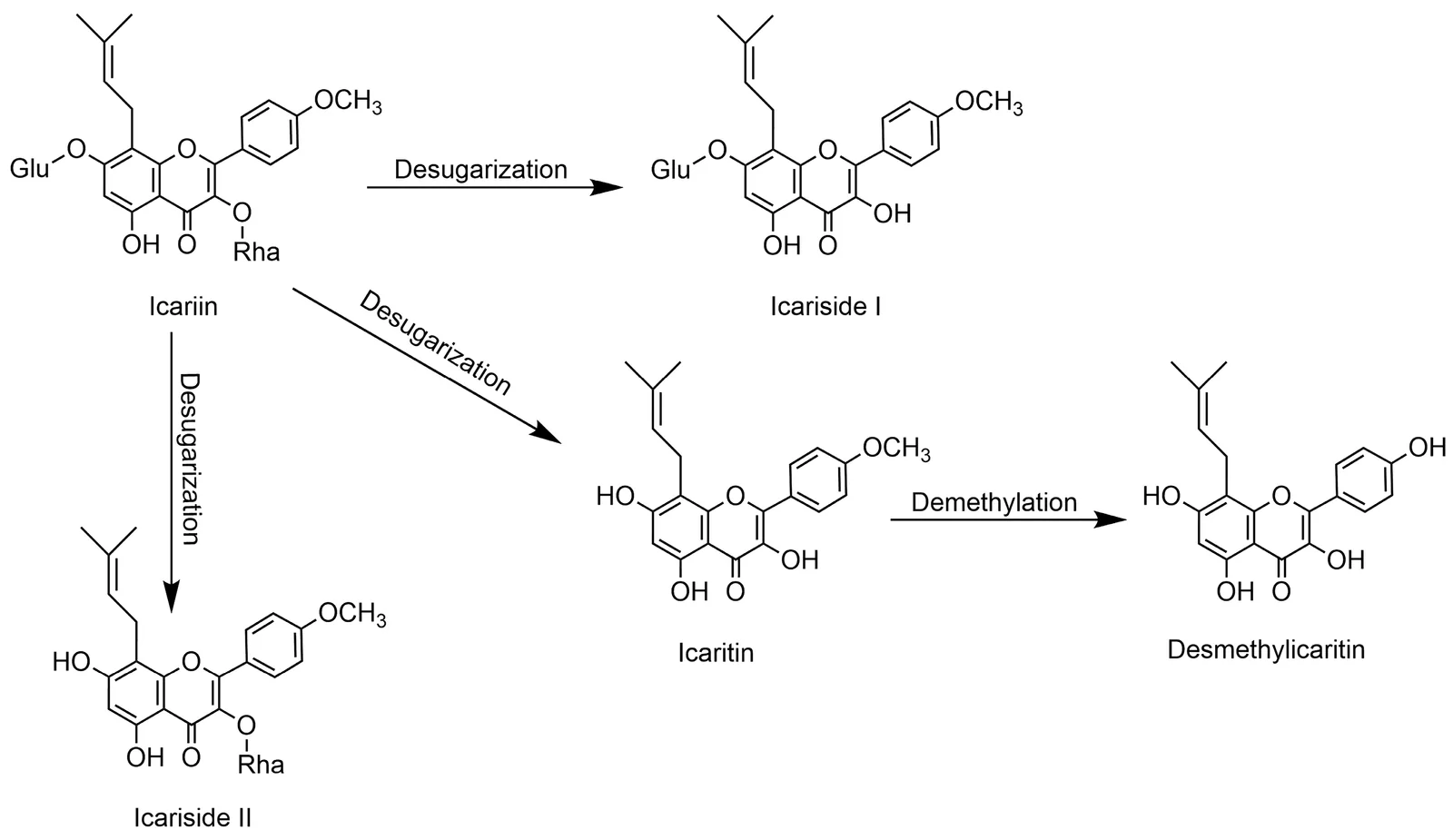
The metabolic pathways of ICA and the structural formula of its metabolites.

ICA is mainly hydrolyzed in the small intestine to ICS I, icariside II (ICS II), ICT and demethylicariin (Figure 4). Scientists studied the metabolism of ICA in different species. It has been reported that ICA was completely transformed into ICS II, ICT and desmethylicaritin in less than 20 h when co-cultured with three intestinal bacteria (*Streptococcus* sp. MRG-ICA-B, *Enterococcus* sp. MRG-ICA-E, and *Blautia* sp. MRG-PMF-1) isolated from human feces [149]. Another experiment studied the metabolism of *Epimedium*-derived flavonoid glycosides (purity of ICA ≥ 83%) in rabbit intestinal flora and discovered that ICA was completely eliminated at 2 h, at which time ICS II reached its maximum peak area and then gradually decreased, while ICT was generated [150]. In rats, the phase I metabolites of ICA are formed in the intestine, including deglycosylated, demethylated, and dehydroxylated metabolites; the phase II metabolites of ICA were related to glucuronides formed in the hepatocyte [151]. The UFLC-TOF/MS technique identified 19 metabolites in the plasma of rats administered with pure ICA (30 mg/kg), including ICS I, ICT, desmethylicaritin, ICS II and other desugarization and dehydrogenation products of ICA [152]. Intragastrically administered ICA was approximately 91.2% converted into ICS II, compared to only 0.4% conversion when administered intravenously [145]. It is speculated that the reason why ICA can be absorbed in large quantities and converted into metabolites is the presence of enzymes in the gastrointestinal tract and intestinal microflora. A previous study demonstrated that ICA exhibited potent inhibition against UGT1A3, while its metabolite ICS II inhibited UGT1A4, UGT1A7, UGT1A9, and UGT2B7, and ICT potently inhibited UGT1A7 and UGT1A9 [153].

### 5.4. Excretion

ICA is basically eliminated after 2~6 h and is not likely to produce accumulated toxicity in animals. A randomized clinical trial confirmed the safety of ICA. Oral administration of *Epimedium* prenylflavonoid extract (containing 6.9% of ICA by weight) did not result in any adverse symptoms and showed no significant pathological changes in liver, blood, and kidney indices [154]. In rats, bile excretion is one of the main ways to eliminate amphoteric, hydrophobic, high molecular weight (relative molecular weight greater than 325) compounds in the body [155]. Due to the water-insoluble characteristics of ICA, it is speculated that the excretion of ICA in the body is mainly through the biliary system [156]. The LC-MS/MS method was established to determine the concentration of ICA in urine after oral administration of EF extract (including ICA 42 mg/kg) in rats. This study found that the excretion process of ICA prototype began during 0–4 h, the average urine cumulative excretion was 5085.46 ng in 36 h, and the ratio of urine cumulative excretion/dosage was 0.43‰, indicating that urine was not the main route of excretion [157]. Another experiment, through qualitative analysis, found that after intragastric administration of 91.8 mg/kg ICA to SD rats, ICA was mainly excreted in the form of glucuronic acid conjugates in bile and urine, and non-glucuronic acid conjugates in feces, and the cumulative excretion of ICA in urine, bile and feces accounted for 0.012‰, 7.0‰, 0.09‰, respectively. The above results showed that ICA was excreted mostly through bile and was also excreted through urine and feces.

## 6. Conclusions and Prospects

As the leading cause of disability and death worldwide, neurological disorders are receiving increasing attention [158]. However, owing to the complexity and uncertain etiology of these diseases, clinical treatment remains largely conservative. Traditional Chinese medicine is considered to hold great potential for new drug development due to its characteristics of “multiple components, multiple targets, and multiple pathways” and its relatively low toxicity. ICA, one of the major active ingredients of natural medicine EF and one of the main components of many proprietary Chinese medicines, has a wide range of pharmacological effects by simultaneously regulating multiple biological pathways and targets. The significant neuroprotective effect of ICA makes it a promising drug candidate for the prevention and treatment of CNS disease.

The main preparation methods for ICA include extraction from the original plant as well as chemical and biological synthesis. Extraction remains the mainstream approach, as it is simple to operate, although the yield is limited by various factors. Chemical synthesis is another feasible route, but it is unsuitable for large-scale production due to its complicated steps and numerous by-products. In contrast, biological synthesis offers several advantages, including environmental friendliness, mild reaction conditions and high yield; moreover, it can make full use of other high-content flavonoids in EF, making it suitable for the industrial production of ICA.

ICA holds great potential for treating CNS diseases. It exerts therapeutic effects through multiple synergistic mechanisms, including neuroprotection, regulation of the release of neurotrophic factors and neurotransmitters, and reduction in the abnormal accumulation of neurotoxic proteins. In addition, the metabolic fate of ICA in vivo affects its potency. After administration, ICA is rapidly absorbed and widely distributed throughout the body and can exert neuroprotective effects across the BBB. Following metabolism, ICA is transformed into several metabolites that retain pharmacological activity. Notably, ICS and ICT, the main metabolites of ICA, are also biologically active and can protect the CNS by inhibiting inflammation or oxidative stress [159,160]. In this process, no significant pathological changes have been observed in organs such as the liver and kidneys.

**Figure 5:**
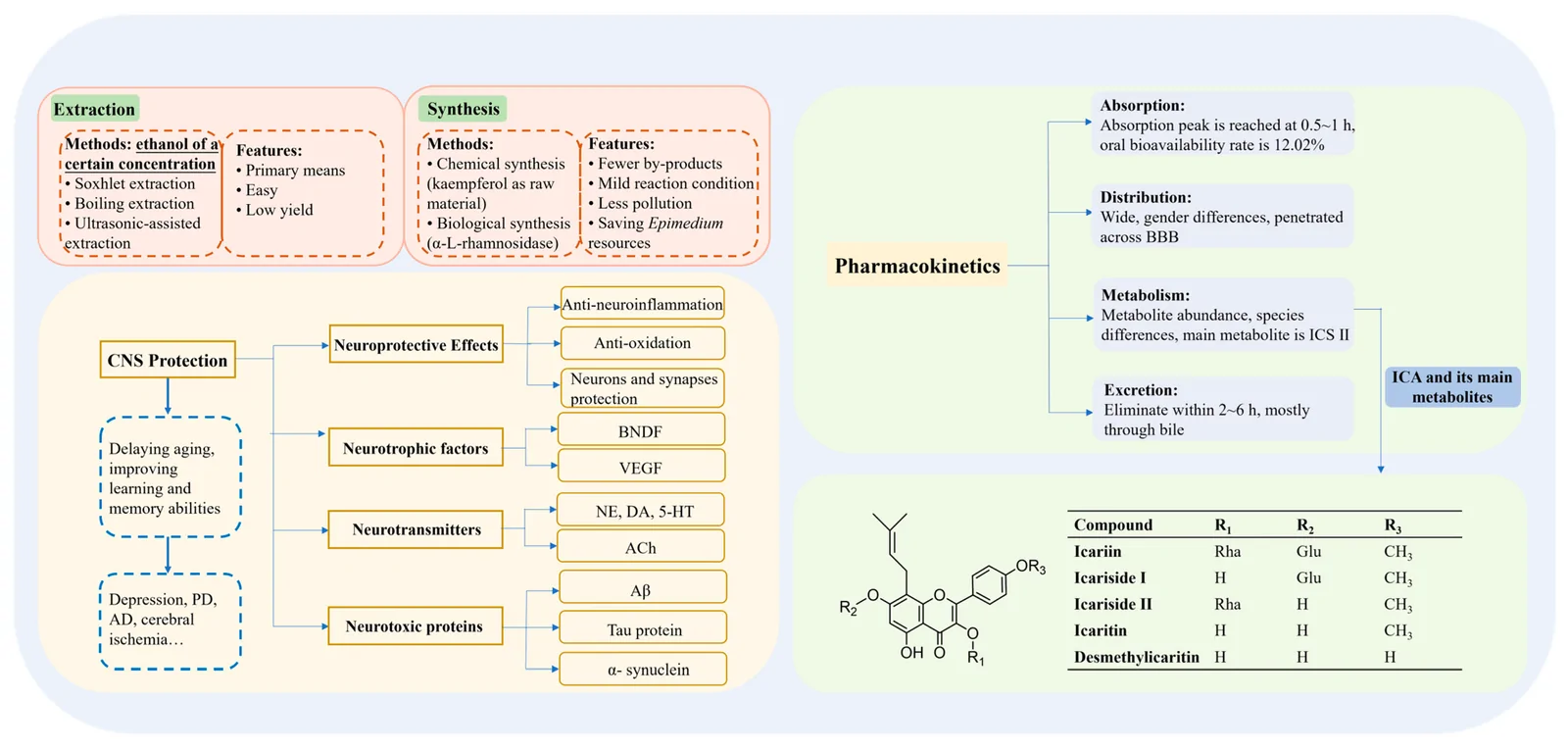
The resources, CNS effects and pharmacokinetics of ICA.

This manuscript (Figure 5) summarizes the preparation methods of ICA, including traditional extraction as well as chemical and biological synthesis, and reviews its pharmacokinetic characteristics, with a focus on the CNS effects of ICA and the underlying mechanisms. We hope this review will provide useful information for further research on ICA.

In conclusion, EF is generally considered one of the most widely used, oldest and most promising traditional Chinese medicines in China, and its main component, ICA, has several effects in different diseases. Apart from its medicinal applications, EF is also used in other fields. For instance, EF extracts are approved as feed additives; they are not only safe, reliable and residue-free, but have also been shown to promote the growth of livestock, prevent cartilage diseases in animals and improve production performance [161].

ICA has now become a promising target for new drug research and development. The latest study found that not only does ICA have a neuroprotective effect, but its metabolites (ICT and ICS II) also hold great promise in the prevention and treatment of neurodegenerative diseases, and they might be promising lead structures in CNS drug development [162]. However, the efficacy of ICA in treating CNS diseases is limited by its poor penetration of the BBB and the lack of precise drug release. Li et al. [163] developed a ROS-responsive selenocysteamine-alginate nanogel (ASeNG-ICA), which can deliver ICA through nasal administration and cross over the BBB. In this way, ICA achieves precise targeting and effectively alleviates behavioral disorders in PD mice. In addition, most current research on ICA remains at the cellular and animal levels; although the results are promising, they are not yet conclusive owing to the lack of clinical trials. This review focuses on the protective effect of ICA on the CNS and its pharmacokinetic parameters, aiming to provide clues for future researchers to explore the clinical applications of ICA.

## Figures and Tables

**Table 1:** The neuroprotective effects of ICA.

Effect	Model	Dose/ Dosing Period	Results	Reference
Anti-neuroinflammation	LPS-induced male SD rats	30, 60, 120 mg/kg 17 days	(a) Decrease escape latency and the searching distance in Morris water maze task; (b) Attenuate the expression of TNF-α, IL-1β and COX-2.	[47]
	LPS-induced BV2 cells	0.01, 0.1 μM 30 min	(a) Suppress microglial activation; (b) Decrease NO, IL-1β and IL-18 production via activation of Nrf2 signaling pathway.	[48]
	LPS-induced primary astrocytes	10, 20 μM 30 min	(a) Down-regulate the expression of TNF-α, IL-1β, iNOS and COX-2; (b) Increase the phosphorylation of ERK1/2 and Akt via IGF-1 receptor signaling pathway.	[49]
Anti-oxidation	SCI male SD rats	35 μM 7 days	(a) Decrease ROS level and MDA content; (b) Increase Sod activity and GSH levels.	[50]
	Aged C57BL/6J male mice	100 mg/kg 15 days	(a) Increase water consumption; (b) Improve the motor learning and coordination abilities; (c) Increase SOD enzymatic activities; (d) Decrease H_2_O_2_ level and MDA content in the serum, brain, liver, and kidney; (e) Activate p-Akt/Nrf2.	[51]
Mitochondria protection	SCI C57BL mice	20, 50 μM 14, 28, 42 days	(a) Enhance motor recovery, restore mitochondrial function through promoting MMP, ATP content, Na-K-ATPase activity, state III respiration rate and RCR; (b) Inhibit the level of mitochondrial apoptotic proteins.	[52]
	Corticosterone-induced primary culture rat hippocampal neuron	0.1, 1, 10 mM 2 h	(a) Improve mitochondrial membrane potential; (b) Inhibit the activation of caspase-3.	[53]
Neuron cell protection	SCI male SD rats	35 μM 7 days	(a) Ameliorate locomotor function and alleviate spinal cord water; (b) Reduce the levels of Bax, caspase- 3 and caspase-9; (c) Up-regulate the expression of Bcl-2.	[50]
	SAMP8 mice	20, 40, 80 mg/kg 3 months	(a) Reduce the spatial memory impairment in a dose-dependent manner; (b) Reverse the decrease in pyramidal layer cells and loss of neurons in Nissl bodies in CA1 region of hippocampus.	[54]
	Aβ_42_-induced primary cortical neuron	40, 80, 160, 320 μg/mL 24 h	(a) Enhance neuronal viability and mortality in a dose-dependent manner; (b) Increase the mRNA and protein levels of cocaine and amphetamine-regulated transcript; (c) Activate MAPK/ERK pathway.	[55]

TNF-α, tumor necrosis factor-α; IL-6, interleukin-6; ROS, reactive oxygen species; MDA, malondialdehyde; SOD, superoxide dismutase; BDNF, brain-derived neurotrophic factor; VEGF, vascular endothelial growth factor; DA, dopamine; NE, norepinephrine; 5-HT, 5-hydroxytryptamine; ACh, acetylcholine; Aβ, amyloid β-protein; IL-1β, interleukin-1 beta; COX-2, cyclooxygenase-2; Nrf2, nuclear factor erythroid 2-related factor 2; iNOS, inducible nitric oxide synthase; ERK1/2, extracellular signal-regulated kinase 1/2; Akt, protein kinase B; p-Akt, phosphorylated protein kinase B; MAPK/ERK, mitogen-activated protein kinase/extracellular signal-regulated kinase.

**Table 2:** In vivo experimental evidence of the beneficial effects of ICA on various diseases.

Disease	Molding Method	Dose (mg/kg)	Pathway/Mechanism	Reference
Depression	CUMS	20, 40	NLRP3 inflammasome signaling pathway	[118]
	social contact stress model	30, 60	HPA axis pathway	[56]
	corticosterone injection	60	Regulate energy, lipid, amino acid and gut microbe metabolic pathways	[119]
	PS—induced offspring depression model	40, 80	Reverse all; These protein expression alterations in HIP and PFC	[120]
	PS—induced offspring depression model	40, 80	Activate the PINK1/Parkin signaling pathway to enhance mitophagy	[121]
AD	water containing aluminum	60, 120	Antioxidant pathway and decrease lipid peroxidation and Aβ_1-40_ levels	[122]
	Aβ_25-35_ injection	30, 60, 120	Suppress β-secretase expression	[123]
	Aβ_25-35_ injection	30, 60, 120	TLR4/MyD88/NF-κB p65 signaling pathway	[124]
	APP/PS1 double-transgenic AD mice	5, 10	Ferroptosis signaling pathway	[125]
	(APP)/(PS1) transgenic mice and C57BL/6J mice; BV-2 microglial cells	100	COX-2-NLRP3 inflammasome-GSDMD axis	[126]
	3 × Tg-AD mice; HT22 cells injured by Aβ_25-35_	30, 60	Wnt/b-catenin pathway	[127]
	ICR mice by injecting of Aβ_1-42_	30, 60, 120	cGAS-STING pathway	[128]
PD	MPTP injection + Ovariectomy	50, 100, 200	PI3K/Akt and MEK/ERK signaling pathways	[28]
Ischemia injury	MCAO	10, 30	NF-κB, PPARα and PPARγ signaling pathway	[129]
	MCAO	25, 50, 100	BDNF and TrkB pathway	[130]
	MCAO	20, 40, 80	NLRP3 inflammasome signaling pathway	[62]
	carotid artery clamping method	30, 80	Antioxidant and anti-inflammation pathway	[60]

CUMS, chronic unpredictable mild stress; NLRP3, NLR family pyrin domain containing 3; HPA, hypothalamic–pituitary–adrenal axis; PS, prenatal stress; MPTP, 1-methyl-4-phenyl-1,2,3,6-tetrahydropyridine; 6-OHDA, 6-hydroxydopamine; LPS, lipopolysaccharide; MCAO, middle cerebral artery occlusion; COX-2, cyclooxygenase-2; GSDMD, gasdermin D; cGAS, cyclic GMP-AMP synthase; STING, stimulator of interferon genes.

**Table 3:** Pharmacokinetic parameters in plasma of ICA as a monomer or drug component.

Species	Drug	Dose (mg/kg)/ Administration	C_max_ (ng/mL)	T_max_ (h)	T_1/2_ (h)	AUC_0-t_ (ng·h/mL)	Reference
Dog	Er-Xian Decoction	0.5 g/kg, i.g.	1.35 ± 1.67	0.75 ± 1.35	-	1.26 ± 1.04	[131]
	*Epimedium* extract	0.5 g/kg, i.g.	2.81 ± 2.61	1.58 ± 3.15	-	4.99 ± 7.71	
Rat	ICA	15 mg/kg, i.v.	13,266.22 ± 5589.44	0.03 ± 0.00	2.25 ± 2.00	1299.44 ± 480.92	[132]
	ICA	50 mg/kg, i.g.	162.4 ± 42.8	1.12 ± 0.31	2.08 ± 0.73	524.6 ± 132.5	[133]
	ICA	10 mg/kg, i.v.	-	-	0.562 ± 0.20	8.70 ± 2.26	[134]
	Jia-Wei-Qi-Fu-Yin	1.5 g/kg, i.g.	3.32 ± 0.56	0.08 ± 0.00	10.71 ± 1.53	11.75 ± 0.30	[135]
		3.0 g/kg, i.g.	16.17 ± 3.79	0.20 ± 0.05	6.72 ± 3.01	21.49 ± 1.23	
		4.5 g/kg, i.g.	30.71 ± 5.40	0.11 ± 0.05	7.88 ± 0.88	31.44 ± 5.29	
	Luhong recipe	ICA 2.75 mg/kg, i.g.	980.00 ± 365.00	0.10 ± 0.16	0.69 ± 0.34	675.00 ± 268.00	[136]
	Gushudan	ICA 155.25 mg/kg, i.g.	24.63 ± 4.67	0.14 ± 0.09	0.38 ± 0.08	15.74 ± 2.53	[137]
	Gushudan capsules	ICA 120 mg/kg, i.g.	236 ± 96	0.21 ± 0.06	0.77 ± 0.20	227 ± 123	[138]
	Shenge Bushen capsules	800 mg/kg, i.g.	530	2.32	3.59	2080	[139]
	Xianlingpi granules	8.1 g/kg, i.g.	48.76 ± 3.98	0.5	28.07 ± 14.69	138.19 ± 45.35	[140]

p.o., oral administration; i.g., intragastric administration; i.v., intravenous administration; C_max_, maximum plasma concentration; T_max_, time to maximum plasma concentration; T_1/2_, elimination half-life; AUC, area under the concentration–time curve.

## Data Availability

No new data were created or analyzed in this study. Data sharing is not applicable to this article.

## Abbreviations

The following abbreviations are used in this manuscript:

ACh	Acetylcholine
AD	Alzheimer’s disease
Akt	Protein kinase B
ATP	Adenosine triphosphate
AUC	Area under the concentration–time curve
Aβ	Amyloid β-protein
BBB	Blood–brain barrier
BDNF	Brain-derived neurotrophic factor
Caspase-1	Cysteine-dependent aspartate-specific protease 1
cGAS	Cyclic GMP-AMP synthase
ChE	Cholinesterase
Cmax	Maximum plasma concentration
CNS	Central nervous system
CNSDs	Central nervous system disorders
COX-2	Cyclooxygenase-2
CUMS	Chronic unpredictable mild stress
DA	Dopamine
EF	Epimedii Folium
ERK	Extracellular signal-regulated kinase
GSDMD	Gasdermin D
GSH	Glutathione
HPA	Hypothalamic–pituitary–adrenal axis
ICA	Icariin
ICSII	Icariside II
ICT	Icaritin
i.g.	Intragastric administration
IGF-1	Insulin-like growth factor 1
IL-18	Interleukin-18
IL-1β	Interleukin-1β
IL-6	Interleukin-6
iNOS	Inducible nitric oxide synthase
i.v.	Intravenous administration
LPS	Lipopolysaccharide
MAPK	Mitogen-activated protein kinase
MCAO	Middle cerebral artery occlusion
MDA	Malondialdehyde
MMP	Mitochondrial membrane potential
MOMP	Mitochondrial outer membrane permeabilization
MPTP	1-methyl-4-phenyl-1,2,3,6-tetrahydropyridine
NE	Norepinephrine
NLRP3	NLR family pyrin domain-containing 3
NO	Nitric oxide
Nrf2	Nuclear factor erythroid 2-related factor 2
OGD/R	Oxygen–glucose deprivation and reoxygenation
PD	Parkinson’s disease
p.o.	Oral administration
PS	Prenatal stress
RCR	Respiratory control ratio
RE	Renal encephalopathy
ROS	Reactive oxygen species
SAMP8	Senescence-accelerated mouse prone 8
SCI	Spinal cord injury
SOD	Superoxide dismutase
STING	Stimulator of interferon genes
T_1/2_	Elimination half-life
T_max_	Time to maximum plasma concentration
TNF-α	Tumor necrosis factor-α
T-OAC	Total antioxidant capacity
TrkB	Tropomyosin receptor kinase B
UAE	Ultrasonic-assisted extraction
VEGF	Vascular endothelial growth factor
5-HT	5-Hydroxytryptamine
